# Widely Targeted Metabolomics Reveals Differences in Secondary Metabolites in Oats With Different Grain Hardness

**DOI:** 10.1002/fsn3.71515

**Published:** 2026-03-18

**Authors:** Daxiao Zhang, Huiyan Liu, Ying Guo, Guanyu Chen, Meilin Chen, Xian Xian, Yuehua Zhang, Bing Han

**Affiliations:** ^1^ Inner Mongolia Agricultural University Hohhot China; ^2^ Key Laboratory of Wheat Germplasm Innovation and Utilization Autonomous Region Higher School Hohhot China; ^3^ Key Laboratory of Grassland Resources of the Ministry of Education Hohhot China; ^4^ Inner Mongolia Autonomous Region Key Laboratory of Biomanufacturing Hohhot China; ^5^ National Center of Pratacultural Technology Innovation (under preparation) Hohhot China

**Keywords:** grain hardness, metabolic pathway, metabolomics, oat, UHPLC–MS/MS

## Abstract

Grain hardness is a crucial factor influencing the processing quality of cereals. This study utilized UHPLC–MS/MS technology to explore novel changes in metabolites associated with grain hardness in the oat cultivars HX317 and HX320. By integrating metabolomics with multivariate statistical methods, we identified 409 secondary metabolites and demonstrated the differences in metabolite profiles between seeds exhibiting varying levels of hardness. The synergistic action of flavonoids cirsilineol (4′,5‐dihydroxy‐3′,6,7‐trimethoxyflavone) and swertiajaponin suggests a potential mechanism for regulating reactive oxygen species accumulation, which may subsequently induce ⍺‐amylase degradation of branched starch through gibberellic acid and abscisic acid pathways, thereby potentially influencing grain hardness. These findings enhanced our understanding of the metabolic mechanisms underlying oat grain hardness and provided a theoretical basis for improving oat processing quality.

## Graphical abstract

UHPLC–MS/MS and metabolomics revealed 409 differentially accumulated secondary metabolites between hard (HX320) and soft (HX317) oat cultivars. Key flavonoids, cirsilineol and swertiajaponin, showed synergistic effects potentially modulating reactive oxygen species. Their interaction may influence α‐amylase‐mediated starch degradation via gibberellic acid and abscisic acid pathways, suggesting a mechanism for grain hardness variation. This metabolic insight advances understanding of oat processing quality regulation.

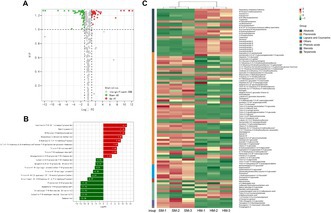

## 1 Introduction

Oats, a monocotyledonous herbaceous plant of the genus *Avena* in the subfamily *Pooideae* of the family *Gramineae*, is a crop cultivated worldwide. Based on the degree of attachment between the lemma and the grain, oats can be classified into two types: naked oats, which have a loose husk, and hulled oats, which have a protective husk (Jenkins and Hanson 1976; Ougham et al. 1996). Grain hardness, defined as the force required to break the grain, is a key trait for market grading and pricing. It affects water absorption capacity, milling yield, damaged starch content, and particle size distribution of flour, ultimately determining the quality of milling and food processing (Feng et al. 2005; Leone and Ferrante 2023). Oat grains are characterized by a markedly soft structure, making them susceptible to damage during harvesting, cleaning, threshing, and dehulling processes (Peltonen et al. 2001). This significantly impacts the processing and edible quality of oats.

Among cereal crops, methods for testing wheat grain hardness have been comprehensively studied, with mechanical methods being the most traditional (Tran et al. 1981). The composition of oat grains is directly associated with their hardness. In the endosperm of cereal grains, approximately 67% of the endosperm lipids are starch lipids, specifically polar lipids (glycolipids and phospholipids), with higher contents in soft wheat than in hard wheat (Haixia 2017). Finnie found that hardness is not associated with the level of neutral lipids in the grain but is closely associated with the surface polar lipids of the washed starch, specifically monogalactosyl diglyceride (MGDG) and digalactosyl diglyceride (DGDG) content (Finnie et al. 2010). β‐glucan and arabinoxylan are structural polysaccharides of the cell wall, contributing to its strength. The content of non‐starch polysaccharides in barley grains is positively correlated with grain hardness (Gamlath et al. 2008). Chiremba found that the high concentration and crosslinking of phenolic acids on the cell walls of the pericarp and aleurone layer are significant. Therefore, phenolic acids may affect the structural properties that influence grain hardness (Chiremba, Rooney, and Beta 2012). Metabolomics, an advanced technological tool, can help us effectively identify differential metabolites and reveal the metabolic processes and regulatory mechanisms of organisms. Among these, secondary metabolites, which act as chemical defensive substances in organisms, exhibit diverse and bioactive characteristics.

This study aimed to explore the metabolic mechanisms underlying oat grain hardness using UHPLC–MS/MS (ultra high performance liquid chromatography/tandem mass spectrometry) metabolomics methods, thereby providing theoretical references for oat breeding efforts.

## 2 Materials and Methods

### 2.1 Plant Materials

To identify the substances affecting the hardness of oat grains, this study selected two cultivars of oats, namely soft oats (HX317) and hard oats (HX320) for metabolomics analysis. The hardness of the grains was calculated based on the relationship between compressive force and time, obtained from the sensor of a texture analyzer during the compression of oat grains. Mature grain samples, harvested in 2021 from field trials conducted in Ulanqab City, Inner Mongolia, China, were utilized in this study. These samples were collected from the Key Laboratory of Germplasm Innovation and Utilization of Triticeae Crops at Inner Mongolia Agricultural University, Hohhot, China. The samples were stored in the dark until needed and exhibited a moisture content of less than 13%. For each sample, three biological replicates were independently analyzed (0.6 g/replicate).

### 2.2 Sample Preparation and Extraction

Biological samples were freeze‐dried by vacuum freeze‐dryer (Scientz‐100F). The freeze‐dried samples were crushed using a mixer mill (MM 400; Retsch) with a zirconia bead for 1.5 min at 30 Hz. 100 mg of lyophilized powder was dissolved in 1.2 mL 70% methanol solution. The samples were kept in a 4°C refrigerator overnight and vortexed for 30 s at 30‐min intervals (6 times total) during this period to ensure complete extraction. Following centrifugation at 12,000 rpm for 10 min at 4°C, the extracts were filtrated (SCAA‐104, 0.22 μm pore size; ANPEL, Shanghai, China, http://www.anpel.com.cn/) before UHPLC–MS/MS analysis.

### 2.3 UHPLC Conditions

The sample extracts were analyzed using an UHPLC‐ESI‐MS/MS system (UHPLC, SHIMADZU Nexera X2, https://www.shimadzu.com.cn/; MS, Applied Biosystems 4500 Q TRAP, https://www.thermofisher.cn/cn/zh/home/brands/applied‐biosystems.html). The analytical conditions were as follows, UHPLC: column, Agilent SB‐C18 (1.8 μm, 2.1 mm × 100 mm); The mobile phase was consisted of solvent A, pure water with 0.1% formic acid, and solvent B, acetonitrile with 0.1% formic acid. Sample measurements were performed with a gradient program that employed the starting conditions of 95% A, 5% B. Within 9 min, a linear gradient to 5% A, 95% B was programmed, and a composition of 5% A, 95% B was kept for 1 min. Subsequently, a composition of 95% A, 5.0% B was adjusted within 1.1 min and kept for 2.9 min. The flow velocity was set as 0.35 mL per minute; The column oven was set to 40°C; The injection volume was 4 μL. The effluent was alternatively connected to an ESI‐triple quadrupole‐linear ion trap (QTRAP)‐MS (Chen et al. 2013).

### 2.4 ESI‐Q Trap‐MS/MS

LIT and triple quadrupole (QQQ) scans were acquired on a triple quadrupole‐linear ion trap mass spectrometer (Q TRAP), AB4500 Q TRAP UHPLC/MS/MS System, equipped with an ESI Turbo Ion‐Spray interface, operating in positive and negative ion mode and controlled by Analyst 1.6.3 software (AB Sciex). The ESI source operation parameters were as follows: ion source, turbo spray; source temperature 550°C; ion spray voltage (IS) 5500 V (positive ion mode)/−4500 V (negative ion mode); ion source gas I (GSI), gas II (GSII), curtain gas (CUR) were set at 50, 60, and 25.0 psi, respectively; the collision‐activated dissociation (CAD) was high. Instrument tuning and mass calibration were performed with 10 and 100 μmol/L polypropylene glycol solutions in QQQ and LIT modes, respectively. QQQ scans were acquired as MRM experiments with collision gas (nitrogen) set to medium. DP and CE for individual MRM transitions was done with further DP and CE optimization. DP/CE optimization used metabolite‐specific standards (BioBioPha/Sigma‐Aldrich) covering all 409 detected metabolites. A specific set of MRM transitions were monitored for each period according to the metabolites eluted within this period (Figures S2–, S6) (Chen et al. 2013, 2009; Fraga et al. 2010; Eriksson et al. 2006).

### 2.5 Metabolite Identification and Data Processing

Metabolite profiling was performed by MetWare Biotechnology (Wuhan, China) using their MetWare Database (MWDB) widely targeted platform. This database contains over 1000 authenticated plant metabolite standards (BioBioPha, Sigma‐Aldrich, ChromaDex; purity ≥ 95%) with validated MRM transitions and retention times. We focused on secondary metabolites relevant to grain hardness, and 409 compounds were retained after matching Q1/Q3 masses (±0.5 Da), retention times (±0.2 min), and MS/MS spectra to MWDB references. This commercial workflow is standard in metabolomics and ensures consistent identification quality across samples.

The value “9” is automatically assigned when no peak can be integrated for a given metabolite in a sample group. It therefore denotes “not detected,” and such cases were treated as qualitative presence/absence differences rather than quantitative fold changes.

### 2.6 PCA

Unsupervised PCA (principal component analysis) was performed by the statistics function prcomp within R 4.3.2 (www.r‐project.org). Prior to PCA analysis, the metabolomics data were preprocessed using UV (Unit Variance) scaling to normalize the data. This standardization method centers each variable to a mean of 0 and scales to a variance of 1 [(value—mean)/standard deviation], ensuring equal weighting of all metabolites regardless of their absolute concentration levels, thereby improving the stability and biological interpretability of the PCA results.

### 2.7 Hierarchical Cluster Analysis and Pearson Correlation Coefficients

The HCA (hierarchical cluster analysis) results of samples and metabolites were presented as heatmaps with dendrograms, while pearson correlation coefficients (PCC) between samples were calculated by the cor function in R and presented as only heatmaps. Both HCA and PCC were carried out by R package ComplexHeatmap v 2.15.4. For HCA, normalized signal intensities of metabolites (unit variance scaling) are visualized as a color spectrum.

### 2.8 Differential Metabolites Selected

Significantly regulated metabolites between groups were determined by VIP ≥ 1 and absolute log_2_FC (fold change) ≥ 1. VIP values were extracted from OPLS‐DA results, which also contain score plots and permutation plots, generated using R package MetaboAnalyst v 5.0. The data was log transformed (log_2_) and mean centered before OPLS‐DA. In order to avoid overfitting, a permutation test (200 permutations) was performed (Thévenot et al. 2015). Significantly regulated metabolites were screened using VIP ≥ 1 and |log_2_FC| ≥ 1 (MetaboAnalyst v5.0, 200‐permutation test).

### 2.9 KEGG Annotation and Enrichment Analysis

Identified metabolites were annotated using the KEGG Compound database (http://www.kegg.jp/kegg/compound), and annotated metabolites were then mapped to the KEGG Pathway database (http://www.kegg.jp/kegg/pathway.html). Pathways with significantly regulated metabolites mapped to were then fed into MSEA (metabolite sets enrichment analysis), and their significance was determined by hypergeometric test's *p*‐values (Kanehisa and Goto 2000; Chong and Xia 2018).

## 3 Results and Discussion

### 3.1 Analysis of Oat Grain Hardness

In our preliminary study, 260 oat accessions were cultivated over three consecutive years at two distinct locations. The grain hardness of harvested kernels was determined using a texture analyzer method, and mean hardness values were subsequently calculated for each accession (Table S2; Hong et al. 2021). From these extensive evaluations across multiple years and locations, the accession exhibiting the highest mean hardness (37.26 ± 2.82 N) was designated the hard material (HM), while the accession exhibiting the lowest mean hardness (18.62 ± 1.93 N) was designated the soft material (SM). The difference in grain hardness between HM and SM was statistically significant (Figure S1).

### 3.2 Metabolomic Analysis of Oat Grains Exhibiting Varying Hardness Levels

**FIGURE 1:**
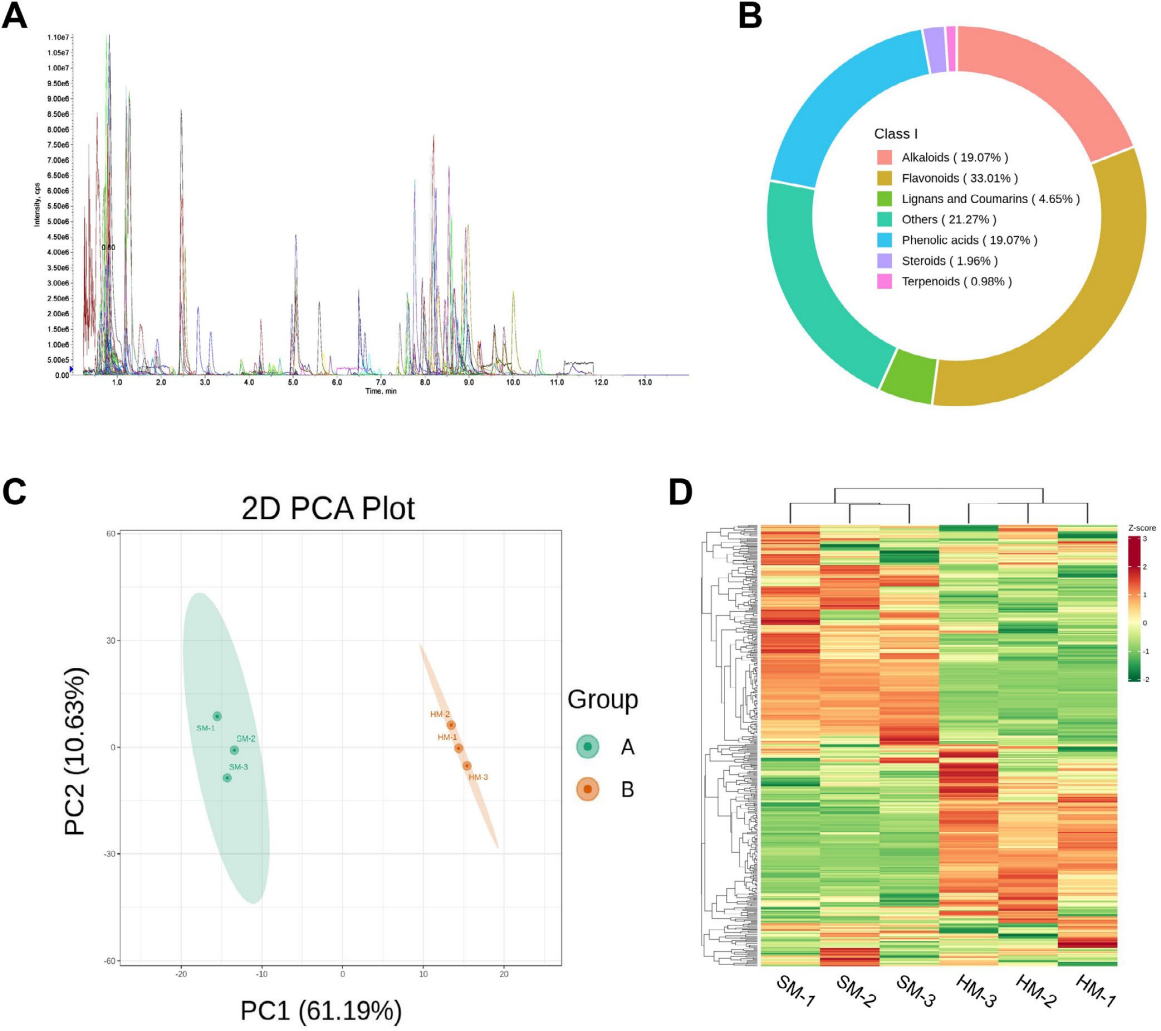
Metabolite profiles of two types of oats. (A), Multi‐peak diagram illustrating MRM metabolite detection. (B), Classification of the 409 metabolites in the two types of oats. (C), Principal component analysis of the metabolic profile of the two types of oats. (D), Hierarchical cluster analysis of the two types of oats. Each type of oat is represented by a column, and each metabolite is displayed in a single row. Red indicates a relatively high abundance of the metabolite, while green indicates a relatively low abundance of the metabolite.

The qualitative and quantitative metabolite analysis of the HM and SM oat grains revealed multiple substances via the MRM mode. Each peak, denoted by different colors, represents a detected metabolite. A total of 409 secondary metabolites were identified, with 403 and 402 secondary metabolites detected in the HM and SM groups, respectively (Figure 1A and Table S3). Among these metabolites, 396 were common to both oat types. The SM oats exhibited six unique metabolites, while the HM oats exhibited seven unique metabolites. These metabolites may contribute to the differences in hardness between oat grains. These results indicate that the HM and SM oats contain a diverse range of metabolites. Principal component analysis (PCA) of the metabolomic data revealed significant differences between the metabolites of the HM and SM oats (Figure 1C). Moreover, cluster analysis elucidated the relative abundance of metabolites at different developmental stages (Figure 1D).

### 3.3 OPLS‐DA Was Performed on Two Groups of Oat Grain Samples Exhibiting Different Hardness Levels

**FIGURE 2:**
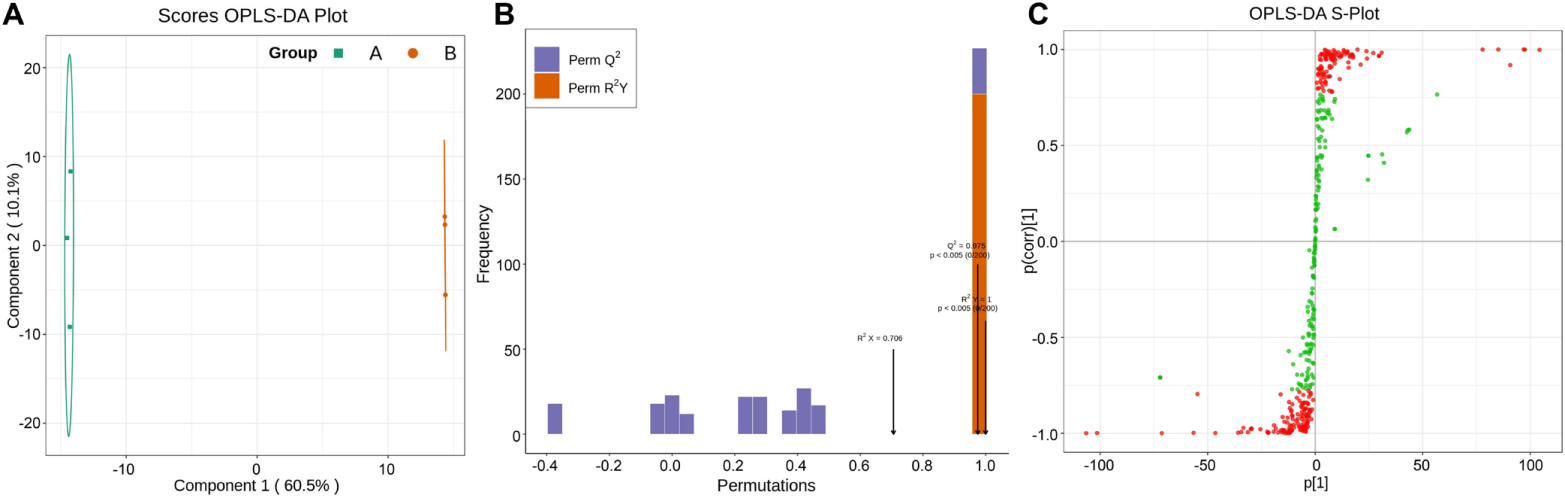
Orthogonal partial least squares discriminant analysis (OPLS‐DA) score plots of two proso oat varieties. (A) OPLS‐DA model plots for the soft (SM) vs. hard (HM) comparison. (B) OPLS‐DA validation diagram. (C) OPLS‐DA score plot.

OPLS‐DA is a multivariate statistical analysis method with supervised pattern recognition capabilities, which effectively eliminates irrelevant influences and selects differential metabolites for study. To investigate the differences between the two groups, the present study utilized OPLS‐DA to evaluate the metabolites in the HM and SM oats. The *R* ^2^X, *R* ^2^Y, and *Q* ^2^ values of the HM and SM oats were 0.722, 1, and 0.976, respectively. The *Q* ^2^ value of the OPLS‐DA model was greater than 50%, indicating good predictive ability (Figure 2B). Permutation tests were performed on the OPLS‐DA model to assess model fitting through 200 iterations. The *p*‐values for PERM *Q* ^2^ and PERM *R* ^2^Y were both less than 0.005, indicating no overfitting in the model. In conclusion, the OPLS‐DA score plot showed clear separation between the groups (Figure 2C), indicating that PCA and OPLS‐DA models can elucidate differences between the HM and SM oats.

### 3.4 Metabolic Characteristics Underlying Oat Grain Hardness

Classification of the 409 secondary metabolites identified earlier resulted in seven categories, based on their properties. These categories included flavonoids (135), phenolic acids (78), alkaloids (78), organic acids (55), lignans and coumarins (19), steroids (8), terpenoids (4), and other metabolites (87) (Figure 1B). Among these, flavonoids, phenolic acids, and alkaloids were the three most abundant metabolite classes.

#### 3.4.1 Flavonoids

**FIGURE 3:**
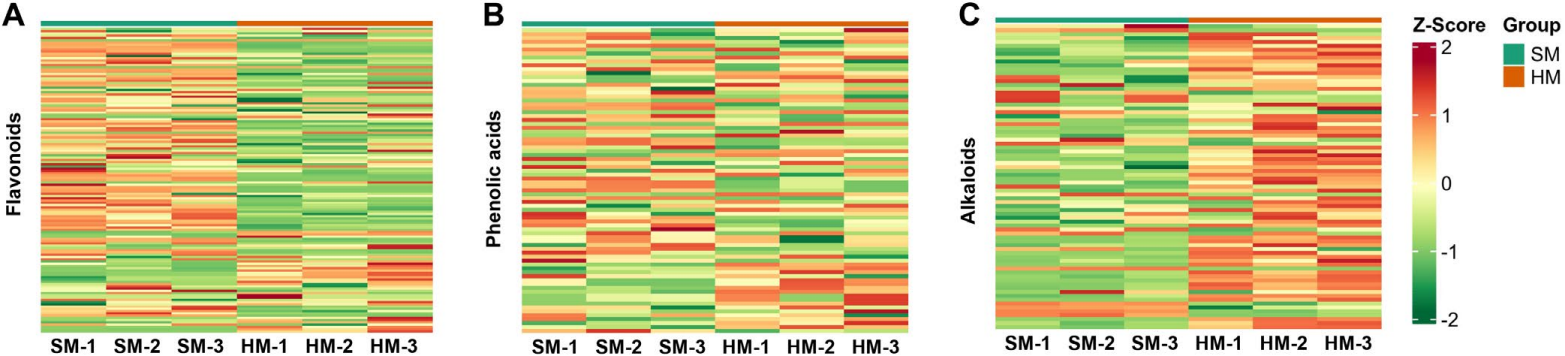
Heatmaps of classified metabolites in the two oat cultivars. Each sample and metabolite is represented by a single column and row, respectively. Red squares indicate an increase in the relative amount of metabolites, and green squares indicate a decrease in the relative amount of metabolites. (A) Flavonoids; (B) phenolic acids; and (C) alkaloids.

In our study, flavonoids were identified as the second most abundant metabolite (Figure 3A). A total of 135 flavonoids were detected, including flavones, flavonols, flavonoid carbonosides, isoflavones, flavanones, flavanonols, chalcones, and dihydroisoflavones. The top 10 flavonoid metabolites included 4′‐hydroxy‐5,7‐dimethoxyflavanone, tricin‐4′‐O‐[β‐guaiacyl‐(9″‐O‐acetyl)glycerol]ether, tricin (5,7,4′‐trihydroxy‐3′,5′‐dimethoxyflavone), and apigenin‐8‐C‐(2″‐xylosyl)glucoside, among others (Table S1). Tricin (5,7,4′‐trihydroxy‐3′,5′‐dimethoxyflavone), a renewable and bioactive polyphenolic compound, is widely distributed in monocots in both free and conjugated forms. It is derived from the secondary metabolism of plants through a biosynthetic pathway analogous to other flavonoids (Nguma et al. 2022). Righetti et al. (2016) extracted tricin from oat husks and found that it exerts an antilipogenic effect (Righetti et al. 2016), thereby reducing grain hardness. Tricin (5,7,4′‐trihydroxy‐3′,5′‐dimethoxyflavone) was expressed at higher levels in the SM oats compared with the HM oats (Jiang et al. 2020).

#### 3.4.2 Phenolic Acids

A total of 78 phenolic acids were identified in this study, among which the top 10 included three derivatives of ferulic acid (FA): 3‐O‐feruloylquinic acid, 1,3‐O‐diferuloylglycerol, and 1,2‐O‐diferuloylglycerol (Figure 3B). FA ranked 16th among the phenolic acids. A significant difference in FA content has been demonstrated between the bran and flour of hard and soft sorghum varieties, with the FA content in hard sorghum flour being twice that of soft sorghum flour (Chiremba, Taylor, et al. 2012). Phenolic acids, as the primary bioactive components in grains, are considered intrinsic biochemical factors that influence the hardness variation of sorghum and maize (Chiremba, Rooney, and Beta 2012). Higher levels of 3‐O‐feruloylquinic acid, 1,3‐O‐diferuloylglycerol, and 1,2‐O‐diferuloylglycerol were observed in HM oats compared to SM oats, suggesting their contribution to increased grain hardness.

#### 3.4.3 Alkaloids

A total of 78 alkaloids were identified in this study, including plumerane, pyridine alkaloids, phenolamine, and other alkaloids. Among these, 5‐aminolevulinic acid was the third most abundant metabolite (Figure 3C). In a study by Gao et al. (2020), the application of 5‐aminolevulinic acid to wheat was found to increase grain dry matter accumulation, thousand‐kernel weight, and grain yield. It was found that the content of 5‐aminolevulinic acid was higher in the HM oats than in the SM oats, indicating that dry matter accumulation in the HM grains was greater than in the SM grains. This is beneficial for grain development and increasing hardness.

### 3.5 Differential Metabolites Between the Two Oat Types

**FIGURE 4:**
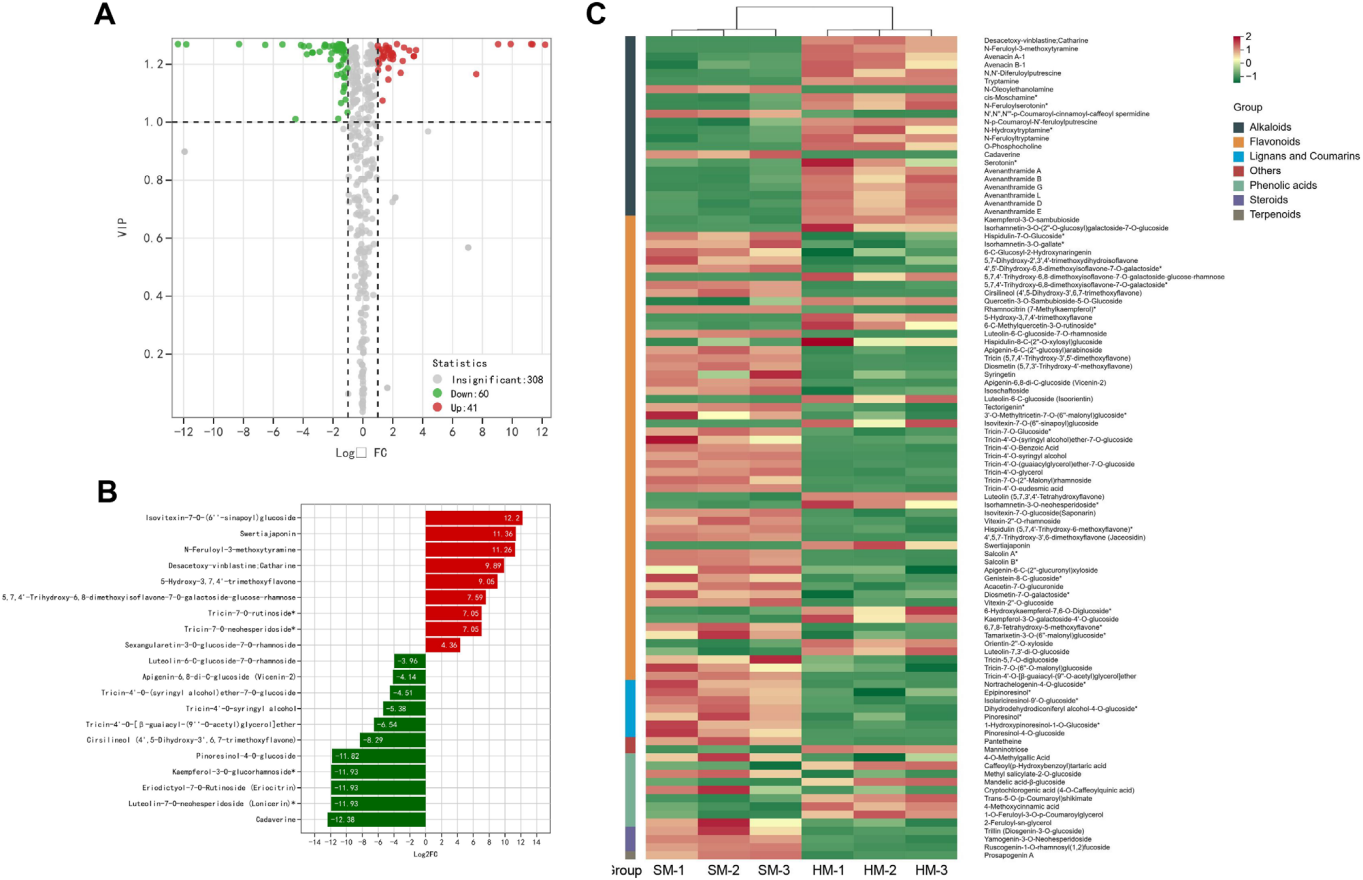
Differentially accumulated metabolites between the soft (SM) and hard (HM) oats. (A) Volcano plot depicting the differential metabolites between the two oat cultivars. (B) Bar graph showing the top 20 differentially expressed metabolites (with log_2_FC) between the SM and HM oats. (C) Clustered heatmap depicting differential metabolite levels between the SM and HM oats. Red squares indicate upregulated metabolites, while green squares indicate downregulated metabolites.

To investigate the differences in metabolites between the HM and SM oats, this study conducted a differential metabolite analysis. The results showed 60 upregulated and 41 downregulated metabolites in the SM oats compared with the HM oats (Figure 4A). Flavonoids were the most abundant differential metabolites, with a total of 57 identified. Specifically, compared with the HM oats, 41 downregulated metabolites and 16 upregulated metabolites were observed in the SM oats (Figure 4B). The metabolite cirsilineol (4′,5‐dihydroxy‐3′,6,7‐trimethoxyflavone) was consistently detected in SM but returned not detected in all HM replicates. Cirsilineol has been shown to induce reactive oxygen species (ROS) accumulation (Wahab et al. 2022). Additionally, compared with the SM oats, swertiajaponin was detected only in HM. As an antioxidant, swertiajaponin can significantly inhibit ROS production and exhibit strong free radical scavenging activity (Moon et al. 2018).

The second most abundant differential metabolites were alkaloids, with a total of 22 identified. Among these, 19 were upregulated and 3 were downregulated. The upregulated metabolites included six FA derivatives: N‐feruloyl‐3‐methoxytyramine, N,N′‐diferuloylputrescine, N‐feruloyltryptamine, N‐feruloylserotonin, cis‐moschamine, and N‐p‐coumaroyl‐N′‐feruloylputrescine. These metabolites showed pronounced enrichment in SM relative to HM, with several detected exclusively in the SM samples. Constance found that the content of FA is directly proportional to the hardness of sorghum grains, which is consistent with our research findings (Kim et al. 2022). The third most abundant differential metabolites were phenolic acid metabolites, with a total of nine identified: five upregulated and four downregulated. The remaining 13 differential metabolites were lignans and coumarins, steroids, others, and terpenoids, with 7, 3, 2, and 1 differential metabolites, respectively. Among these, 1 was upregulated and 12 were downregulated. These secondary metabolites may play various roles in oat grain hardness.

### 3.6 Metabolic Pathway Analysis

**FIGURE 5:**
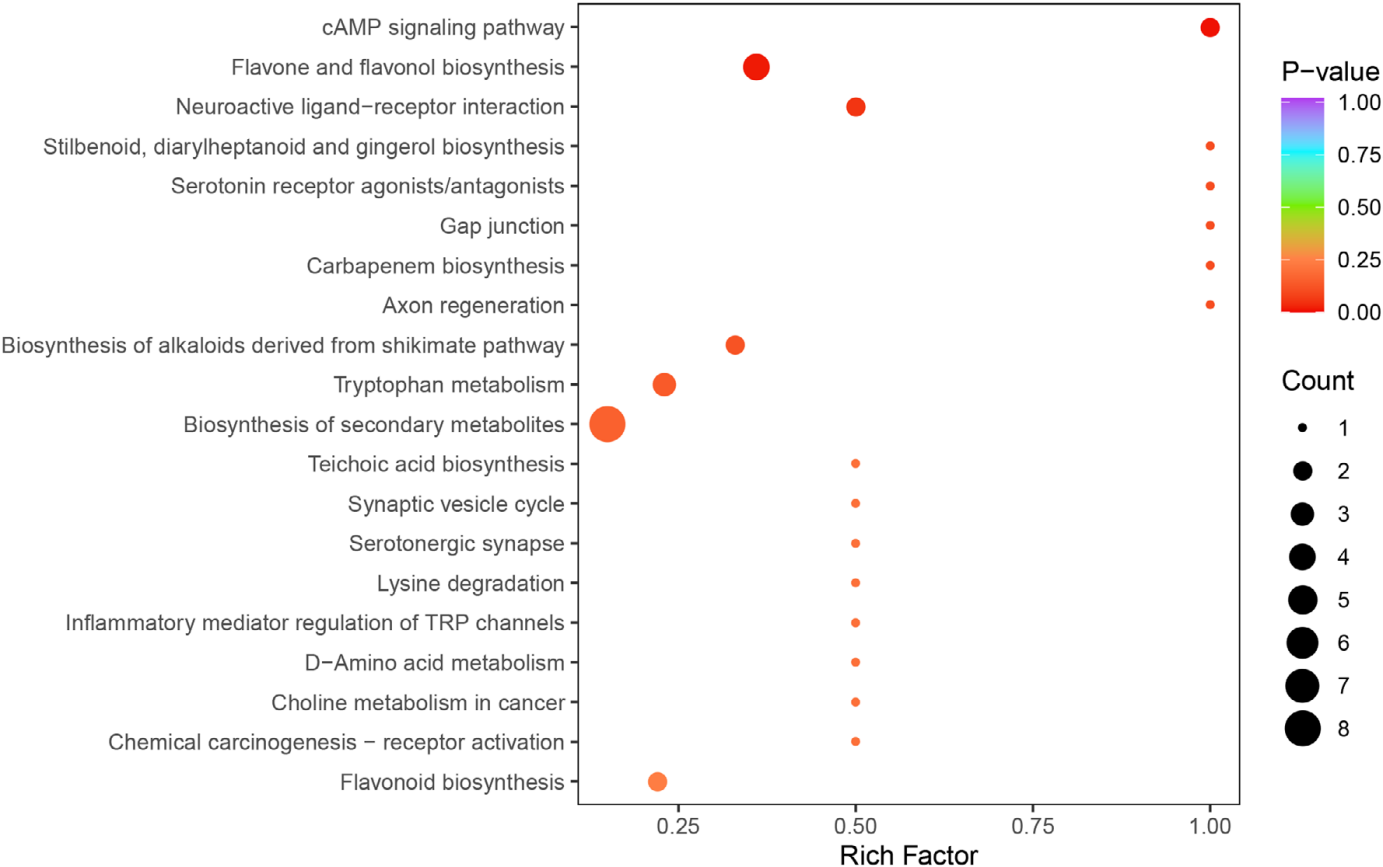
Bubble plot showing the differential enrichment of KEGG pathways between the soft (SM) and hard (HM) oats. Each bubble in the plot represents a metabolic pathway whose abscissa and bubble size jointly indicate the magnitude of the impact factors of the pathway. A greater bubble size indicates a greater impact factor. The bubble colors represent the *p*‐values of the enrichment analysis, with darker colors denoting a higher degree of enrichment.

To further explore pathways potentially associated with grain hardness, KEGG pathway analysis was performed on the identified metabolites. Among the 409 metabolites detected, 119 were associated with KEGG and mapped to 62 metabolic pathways. Significantly enriched pathways included flavonoid and flavonol biosynthesis (ko00944) (Figure 5), with a *p*‐value of less than 0.05, indicating substantial differences in the relative content of flavonoids and flavonols between the HM and SM oats.

Kim et al. (2022) discovered that the gene *OsCOP1* regulates flavonoid biosynthesis and embryonic development (Lin et al. 2023). Lin et al. (2023) found that gramineous plants accumulate a unique series of flavonoids, which contribute to defense responses, fertility, pigment deposition, and cell wall lignification (Zafra et al. 2016). Wahab and Moon investigated the effects of the flavonoids cirsilineol and swertiajaponin on the accumulation of ROS. They found that cirsilineol promotes ROS accumulation, while swertiajaponin significantly inhibits ROS accumulation (Wahab et al. 2022; Moon et al. 2018). ROS, generated during metabolism, play various roles in plants, including involvement in stress responses, defense mechanisms, and signal transduction, regulated by multiple enzyme and non‐enzyme systems (Zafra et al. 2016). Therefore, the presence of ROS has become a key indicator in plant physiology, demonstrating its multifunctionality and significant impact on growth and development.

Suriyasak found that during the rice filling stage, the accumulation of ROS increases the expression of genes involved in gibberellic acid (GA) biosynthesis and abscisic acid (ABA) catabolism. This leads to the induction of α‐amylase, resulting in starch degradation and the production of chalkiness (Suriyasak et al. 2017). Furthermore, Nielsen revealed that the SBS2 binding site of barley α‐amylase is crucial in the branching starch degradation pathway, regulating the degradation of branching starch (Nielsen et al. 2012). Previously, our team determined the content of amylose and amylopectin in different materials and found a significant positive correlation between the hardness of oat grains and the content of amylopectin (An 2022). Therefore, these findings suggest that the contrasting levels of cirsilineol and swertiajaponin in SM material coregulate ROS accumulation. This, in turn, regulates GA synthesis and ABA metabolism, inducing the degradation of branching starch by α‐amylase and resulting in softer grains. In the HM material, this regulatory process is the opposite, leading to higher hardness of the HM material.

## 4 Conclusion

This study presents a novel exploration of the metabolic changes underlying oat grain hardness based on UHPLC–MS/MS technology. A total of 409 secondary metabolites were identified, demonstrating differences in metabolites between grains of varying hardness as well as unique metabolites in each type. Previous studies have shown that the flavonoid tricin and its derivatives significantly impact grain hardness. KEGG analysis revealed that the synthesis of flavonoids and flavonols is a crucial metabolic pathway affecting oat grain hardness. Importantly, our data combined with prior mechanistic evidence (Wahab et al. 2022; Moon et al. 2018; Zafra et al. 2016) suggest a potential model where cirsilineol and swertiajaponin synergistically regulate ROS accumulation, which may subsequently induce α‐amylase‐mediated degradation of branched starch through GA and ABA pathways, thereby potentially influencing grain hardness. These findings enhance our understanding of the metabolic mechanisms underlying oat grain hardness and provide hypotheses and theoretical references for improving oat processing quality, though functional validation of this pathway remains an essential future direction.

## Author Contributions

**Daxiao Zhang:** conceptualization (equal), data curation (lead), formal analysis (lead), investigation (lead), methodology (equal), project administration (equal), resources (equal), software (equal), supervision (equal), validation (equal), visualization (lead), writing – original draft (lead), writing – review and editing (lead). **Huiyan Liu:** formal analysis (supporting), methodology (lead), supervision (lead). **Ying Guo:** data curation (lead), visualization (equal). **Guanyu Chen:** project administration (equal), resources (lead). **Meilin Chen:** data curation (equal), supervision (supporting). **Xian Xian:** methodology (supporting), supervision (supporting). **Yuehua Zhang:** writing – review and editing (supporting). **Bing Han:** conceptualization (equal), data curation (equal), formal analysis (equal), funding acquisition (lead), investigation (equal), methodology (equal), project administration (equal), resources (lead), software (equal), supervision (lead), validation (lead), visualization (equal), writing – original draft (supporting), writing – review and editing (supporting).

## Funding

This work was supported by the Central Guiding Local Science and Technology Development Fund Project of Inner Mongolia Department of Science and Technology, 2022ZY0047, the Innovative Technology System for Oat and Quinoa Industry in Inner Mongolia Autonomous Region, the Key Lab of Germplasm Innovation and Utilization of Triticeae Crop at Universities of Inner Mongolia Autonomous Region construction funds, the National Key Research and Development Program, 2022YFE0119800, National Technology Innovation Center for Prataculture Project: Special fund for innovation platform construction (CCPTZX2024GJ03). [Correction added on 25 March, 2026, after first online publication: The Funder name “Institution: National Center of Pratacultural Technology Innovation (under preparation) Project: Special fund for innovation platform construction (CCPTZX2024GJ03)” is revised to “National Technology Innovation Center for Prataculture Project: Special fund for innovation platform construction (CCPTZX2024GJ03)” in this version.]

## Conflicts of Interest

The authors declare no conflicts of interest.

## Supporting information


**Figure S1:** Box plot of grain hardness distribution in HM (HX320) and SM (HX317) oat cultivars. *** *p* < 0.001 indicates significant difference by Student's *t*‐test.

**Figure S2:** TIC Overlay Plots of QC Samples (Negative Ionization Mode).

**Figure S3:** TIC Overlay Plots of QC Samples (Positive Ionization Mode).

**Figure S4:** Inter‐group CV Distribution Plots of Metabolites.

**Figure S5:** Integrated Metabolite Quantification with Calibration (Negative Ion Mode).

**Figure. S6** Integrated Metabolite Quantification with Calibration (Positive Ion Mode).

**Table S1:** The top 10 substances details of three major categories metabolites in SM and HM.

**Table S2:** Hardness values of 260 core germplasm materials of naked oats in 3 years and 2 fields.

**Table S3:** Metabolites Exclusively Present in SMHM Groups.

**Table S4:** ALL_sample_data.

## Data Availability

The data that support the findings of this study are available from the corresponding author upon reasonable request.
