# Supplementary figures and images for: Widely Targeted Metabolomics Reveals Differences in Secondary Metabolites in Oats With Different Grain Hardness
Source: Food Sci Nutr. 2026 Mar 18;14(3):e71515. doi: 10.1002/fsn3.71515 (PMC13093555; doi:10.1002/fsn3.71515)

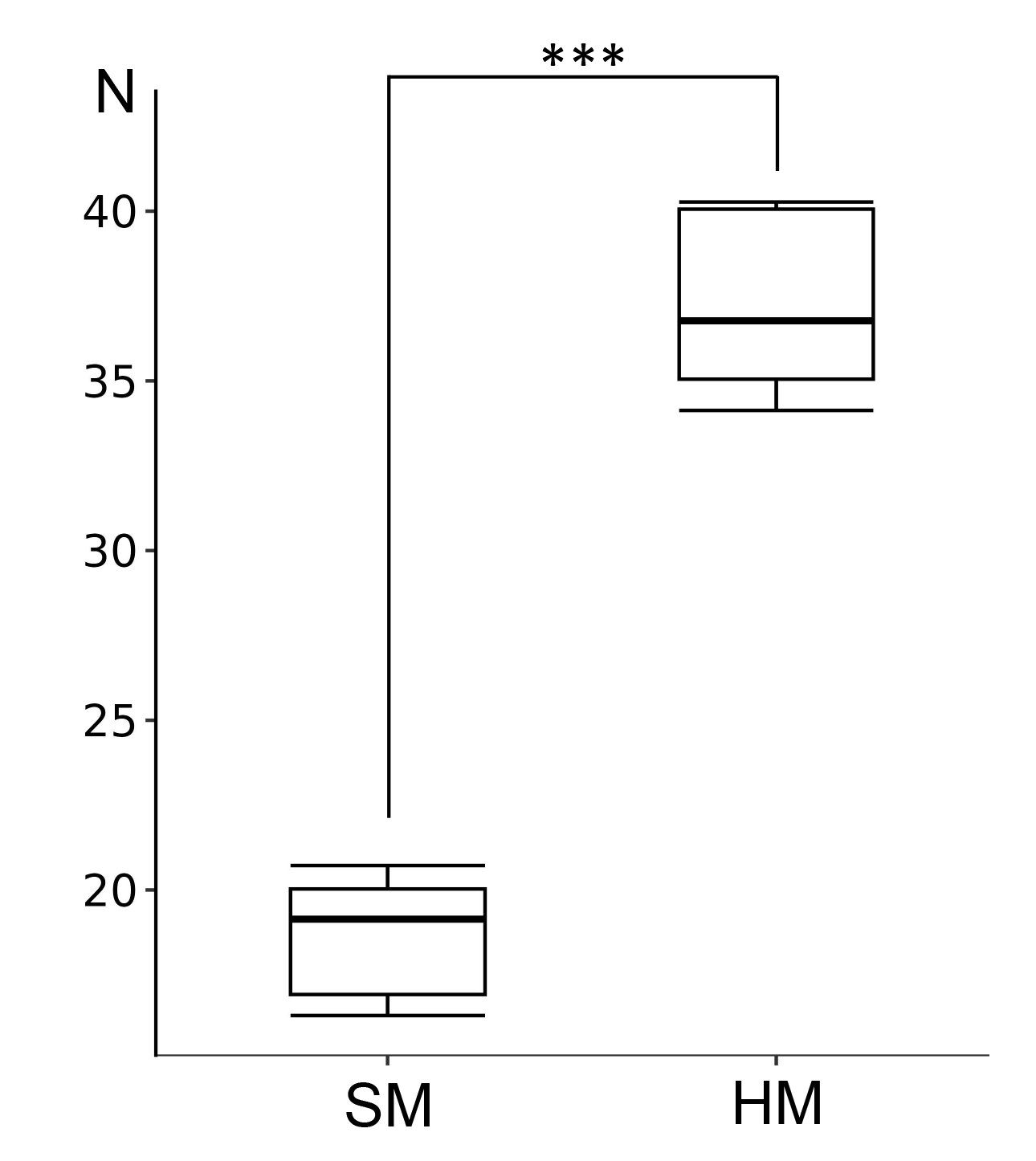

Supplement: Supplementary file 1 — Figure S1: Box plot of grain hardness distribution in HM (HX320) and SM (HX317) oat cultivars. *** p < 0.001 indicates significant difference by Student's t‐test. [file FSN3-14-e71515-s003.tif]

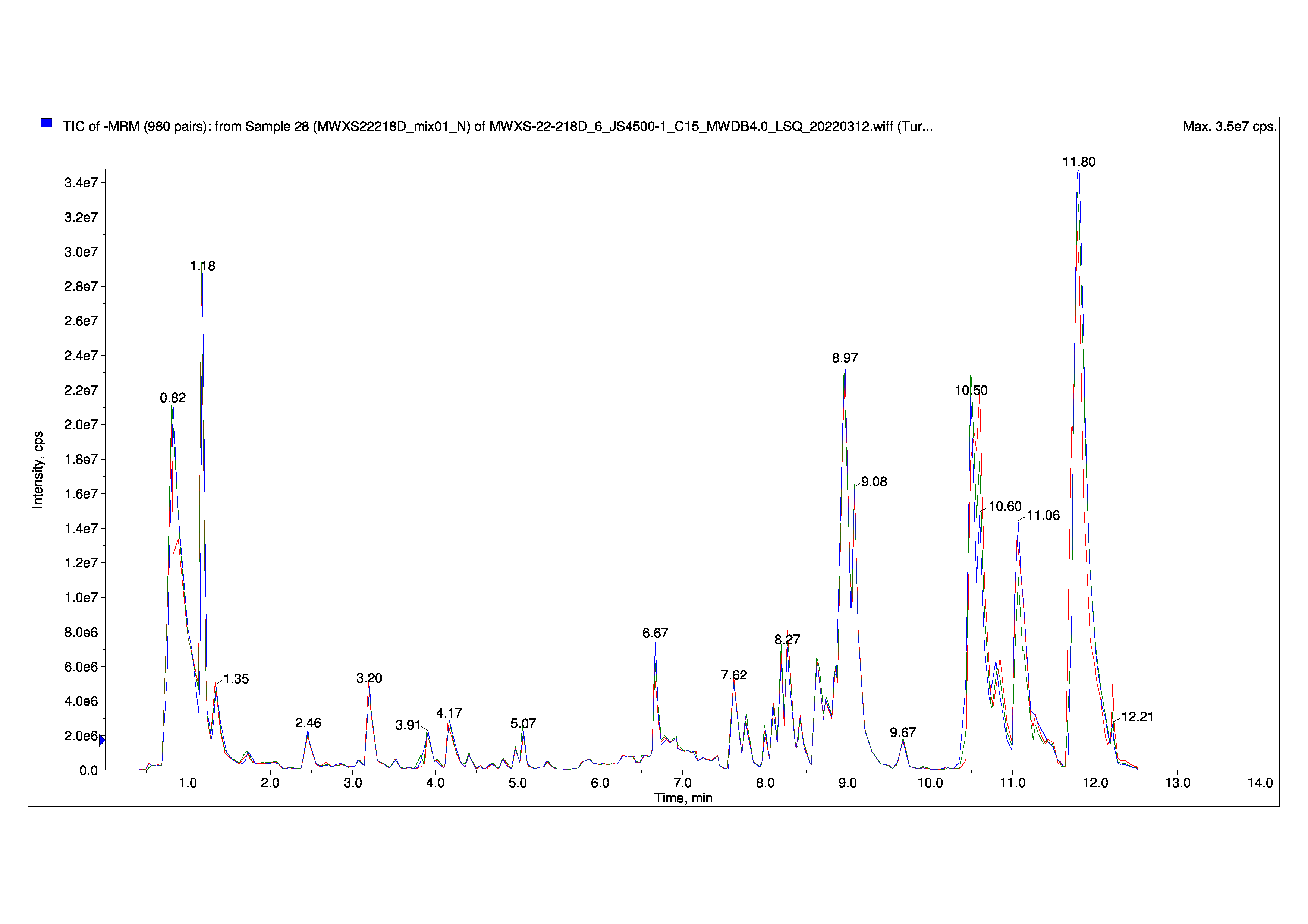

Supplement: Supplementary file 2 — Figure S2: TIC Overlay Plots of QC Samples (Negative Ionization Mode). [file FSN3-14-e71515-s002.png]

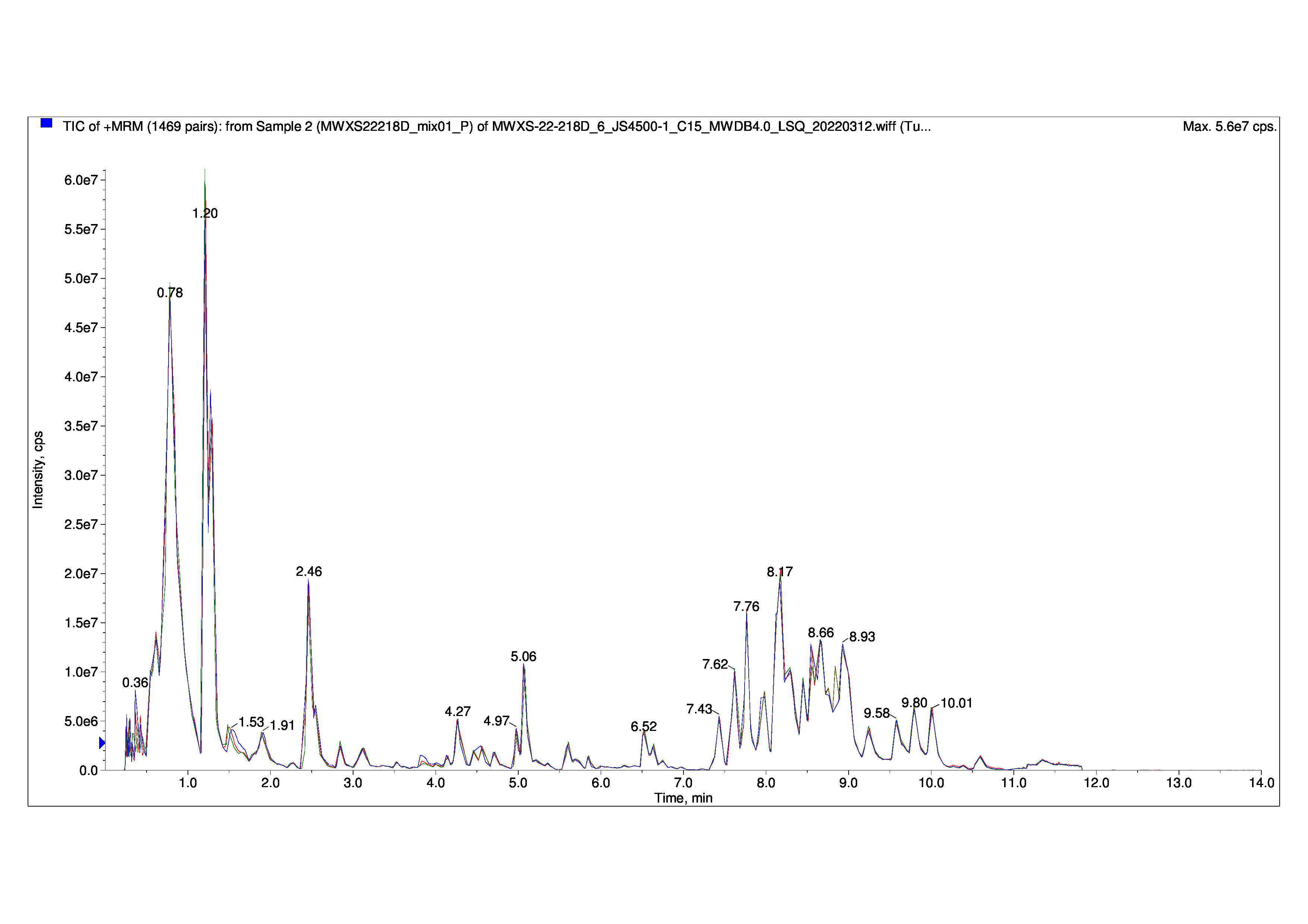

Supplement: Supplementary file 3 — Figure S3: TIC Overlay Plots of QC Samples (Positive Ionization Mode). [file FSN3-14-e71515-s009.png]

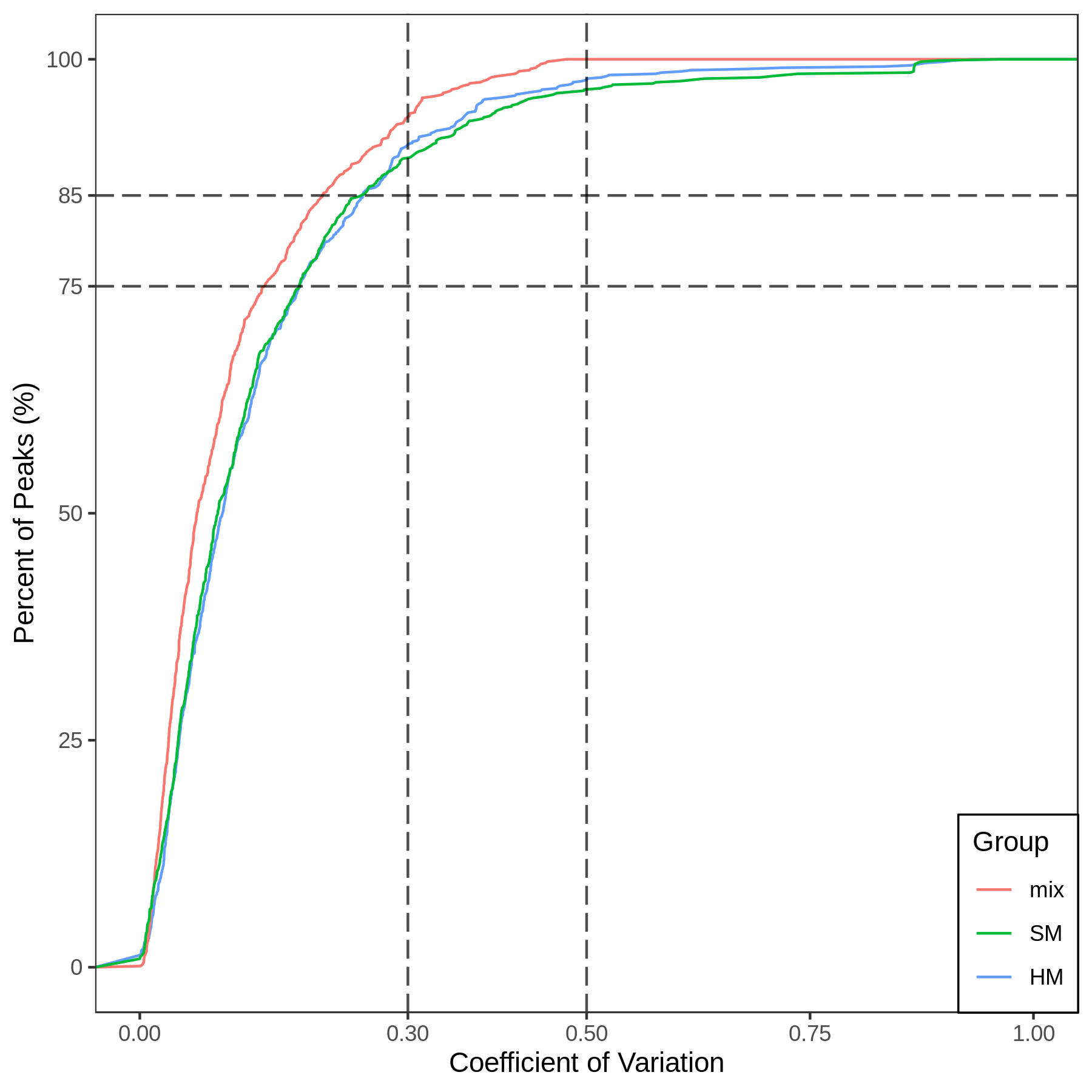

Supplement: Supplementary file 4 — Figure S4: Inter‐group CV Distribution Plots of Metabolites. [file FSN3-14-e71515-s005.png]

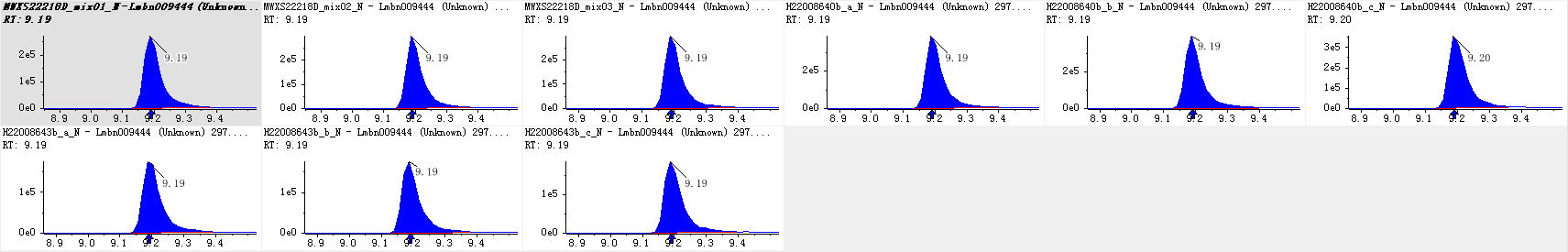

Supplement: Supplementary file 5 — Figure S5: Integrated Metabolite Quantification with Calibration (Negative Ion Mode). [file FSN3-14-e71515-s006.png]

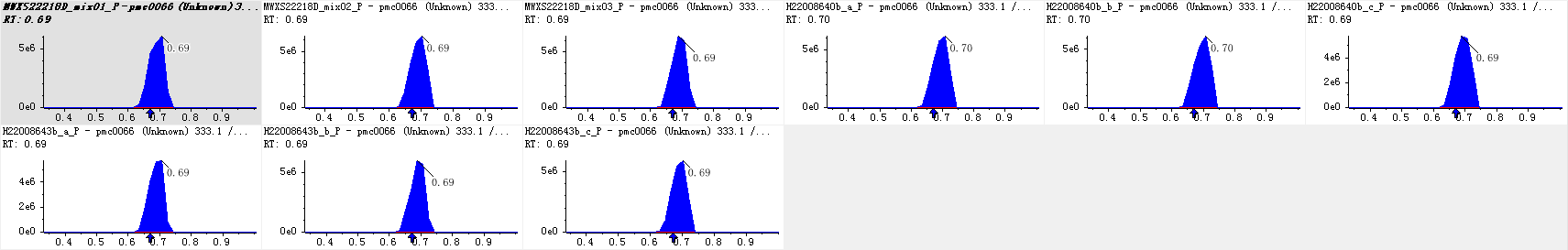

Supplement: Supplementary file 6 — Figure. S6 Integrated Metabolite Quantification with Calibration (Positive Ion Mode). [file FSN3-14-e71515-s010.png]
